# Root Hair Mutations Displace the Barley Rhizosphere Microbiota

**DOI:** 10.3389/fpls.2017.01094

**Published:** 2017-06-26

**Authors:** Senga Robertson-Albertyn, Rodrigo Alegria Terrazas, Katharin Balbirnie, Manuel Blank, Agnieszka Janiak, Iwona Szarejko, Beata Chmielewska, Jagna Karcz, Jenny Morris, Pete E. Hedley, Timothy S. George, Davide Bulgarelli

**Affiliations:** ^1^Plant Sciences, School of Life Sciences, University of Dundee Dundee, United Kingdom; ^2^Department of Genetics, University of Silesia in Katowice Katowice, Poland; ^3^Scanning Electron Microscopy Laboratory, University of Silesia in Katowice Katowice, Poland; ^4^Cell and Molecular Sciences, The James Hutton Institute Dundee, United Kingdom; ^5^Ecological Sciences, The James Hutton Institute Dundee, United Kingdom

**Keywords:** rhizosphere, microbiota, plant–microbe interactions, root hairs, barley

## Abstract

The rhizosphere, the thin layer of soil surrounding and influenced by plant roots, defines a distinct and selective microbial habitat compared to unplanted soil. The microbial communities inhabiting the rhizosphere, the rhizosphere microbiota, engage in interactions with their host plants which span from parasitism to mutualism. Therefore, the rhizosphere microbiota emerges as one of the determinants of yield potential in crops. Studies conducted with different plant species have unequivocally pointed to the host plant as a driver of the microbiota thriving at the root–soil interface. Thus far, the host genetic traits shaping the rhizosphere microbiota are not completely understood. As root hairs play a critical role in resource exchanges between plants and the rhizosphere, we hypothesized that they can act as a determinant of the microbiota thriving at the root–soil interface. To test this hypothesis, we took advantage of barley (*Hordeum vulgare*) mutant lines contrasting for their root hair characteristics. Plants were grown in two agricultural soils, differentiating in their organic matter contents, under controlled environmental conditions. At early stem elongation rhizosphere specimens were collected and subjected to high-resolution 16S rRNA gene profiling. Our data revealed that the barley rhizosphere microbiota is largely dominated by members of the phyla Bacteroidetes and Proteobacteria, regardless of the soil type and the root hair characteristics of the host plant. Conversely, ecological indices calculated using operational taxonomic units (OTUs) presence, abundance, and phylogeny revealed a significant impact of root hair mutations on the composition of the rhizosphere microbiota. In particular, our data indicate that mutant plants host a reduced-complexity community compared to wild-type genotypes and unplanted soil controls. Congruently, the host genotype explained up to 18% of the variation in ecological distances computed for the rhizosphere samples. Importantly, this effect is manifested in a soil-dependent manner. A closer inspection of the sequencing profiles revealed that the root hair-dependent diversification of the microbiota is supported by a taxonomically narrow group of bacteria, with a bias for members of the orders Actinomycetales, Burkholderiales, Rhizobiales, Sphingomonadales, and Xanthomonadales. Taken together, our results indicate that the presence and function of root hairs are a determinant of the bacterial community thriving in the rhizosphere and their perturbations can markedly impact on the recruitment of individual members of the microbiota.

## Introduction

The rhizosphere, the thin layer of soil tightly adhering to plant roots and influenced by plant growth and development, represents an environment whose chemical and physical properties are markedly distinct from unplanted soil (Cardon and Gage, 2006). The rhizosphere defines the interface between plant roots and soil and, as such, is the site of transfer of most mineral elements and water from the terrestrial to the biological sphere, with implications for biogeochemical and hydrological cycles on a global scale (White et al., 2013). In cereals, the rhizosphere, whose operational definition often coincides with the rhizosheath, facilitates mineral and water exchanges among plants, microbes, and the soil (McCully, 1999). For instance, in barley (*Hordeum vulgare*), plants capable of forming a consistent rhizosphere (i.e., exceeding 50 g soil/g roots) cope more efficiently with limitations in the availability of essential elements such as phosphorus (George et al., 2014). Likewise, wheat (*Triticum aestivum*) plants with a large rhizosphere successfully thrive under soil stress conditions, such as acidity and aluminum toxicity (Delhaize et al., 2012; James et al., 2016).

At the same time, the rhizosphere represents a distinct microhabitat characterized by enhanced microbial activity compared to unplanted soil (Kent and Triplett, 2002; Ofek et al., 2014). In turn, the bacterial communities inhabiting the rhizosphere, the rhizosphere bacterial microbiota, establish interactions with plant roots which include parasitism, mutualism, and commensalism (Schlaeppi and Bulgarelli, 2015). For instance, it has been demonstrated that individual members of the rhizosphere microbiota are capable of promoting plant performance by, predominantly, enhancing both mineral acquisition from soil (Terrazas et al., 2016) and strengthening pathogen protection (Berendsen et al., 2012). Therefore, deciphering the molecular mechanisms modulating rhizosphere formation and functioning is emerging as a key area of investigation for sustainable crop production.

Predominant among the plant-derived mechanisms shaping the rhizosphere is the process of rhizodeposition whereby plants release, through passive and controlled mechanisms, a plethora of organic compounds in the vicinity of their roots (Nguyen, 2003). This process not only modifies the chemical and physical composition of the rhizosphere, but is also considered a major determinant of its inhabiting microbiota, by providing the soil biota with organic substrates for microbial multiplication (Dennis et al., 2010; Bulgarelli et al., 2013). For instance, variation in rhizodeposition patterns among different barley genotypes coincides with distinct metabolic capabilities executed by their microbiotas (Mwafulirwa et al., 2016), providing an example of host genetic-mediated control of the rhizodeposition impacting on the microbial communities thriving at the root–soil interface.

Root hairs, tubular outgrowths of the root epidermis, play a critical role both in the acquisition of scarcely mobile soil minerals and in rhizodeposition (Gahoonia et al., 1997; Brown et al., 2012). In cereals, root hairs are a major determinant of both rhizosphere formation, i.e., the proportion of soil modified by the roots (George et al., 2014; Delhaize et al., 2015) and functioning, i.e., the metabolic reactions taking place at the root–soil interface (Pausch et al., 2016). Interestingly, in grasses, root hairs also define an evolutionarily conserved site for bacterial colonization. For instance, the beneficial bacterium *Pseudomonas* sp. DSMZ 13134 efficiently colonizes the root hairs of either soil- or quartz sand-grown barley seedlings (Buddrus-Schiemann et al., 2010). Likewise, in finger millet (*Eleusine coracana*), a panicoid grass belonging to the Chloridoideae subfamily (Kellogg, 1998), root hairs have recently been identified as the site of development of multilayered microcolonies of the beneficial bacterium *Enterobacter* sp. M6 (Mousa et al., 2016). However, it is fundamentally unclear whether, and how, root hairs can act as a determinant of the bacterial communities thriving outside of the root corpus in the rhizosphere.

In this study, we used barley as an experimental model to gain novel insights into the role played by root hairs in shaping the rhizosphere microbiota. In particular, we compared the bacterial communities thriving in association with two barley varieties with fully developed root hairs, Karat and Dema, and their backcrossed inbred lines either lacking root hairs, designated *rhl1.a*, or whose root hair development was interrupted at an early stage, shortly after bulge formation, designated *rhp1.b* (Janiak and Szarejko, 2007). Furthermore, to take into account the relationships between plant- and soil-mediated recruitment cues of the rhizosphere microbiota, we performed our experiments in two distinct agricultural soils representative of barley growing areas. Using high-throughput sequencing and computational approaches, we demonstrated that root hairs are a determinant of the bacterial community inhabiting the rhizosphere and that perturbations in their development can markedly impact on the recruitment of individual members of the microbiota.

## Materials and Methods

### Root Hair Morphology Imaging

Seedlings of mutants and parent varieties were grown in aeroponic conditions. First, seeds were surface-sterilized and left overnight at 4°C to start germination. The following day, they were transferred to glass tubes filled with wet cotton bungs and then stuck to the second tube with parafilm. After 5 to 7 days, root hair zones were analyzed with Stemi 2000-C (Zeiss) stereoscopic microscope and AxioVision LE (Carl Zeiss) software. For scanning electron microscopy (SEM), the 1 cm segments from the root differentiation zone of 7-day-old seedlings were analyzed. The segments were fixed in 3% glutaraldehyde in a 0.1 M sodium phosphate buffer, pH 7.2 for 24 h. After that time the segments were washed three times with the same buffer and post-fixed in 2% osmium tetroxide in a phosphate buffer for 2 h. After subsequent triple washing with a 0.1 M sodium phosphate buffer, pH 7.2, samples were dehydrated through an ethyl alcohol series (50, 60, 70, 80, 90, 95, and 100%, 10 min at each step). The samples were dried in a Critical Point Pelco-CPD2 apparatus using carbon dioxide and then mounted on aluminum stubs with double-sided tape, sputter coated with gold in a Pelco SC-6 sputter coater and viewed and photographed using a Tesla BS 340 scanning electron microscope at 20 kV. Fomapan Type 400/120 film was used to record the images.

### Plant Genotypes and Growth Conditions

**FIGURE 1:**
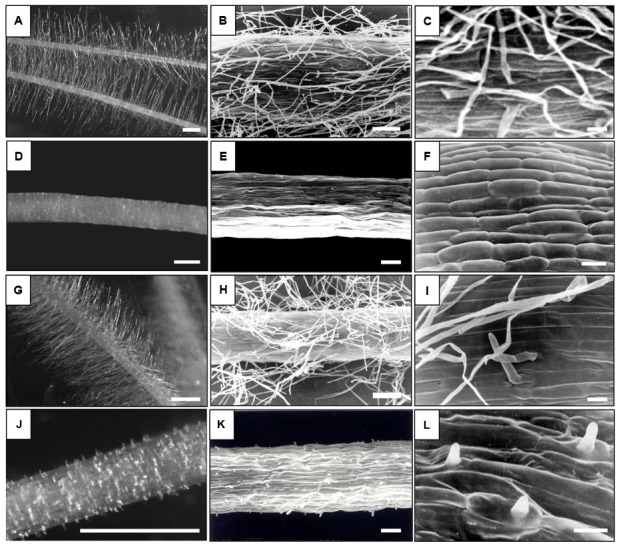
Root hair morphology of the genotypes used in this study. **(A–C)** – ‘Karat,’ **(D–F)** *rhl1*.a mutant, **(G–I)** – ‘Dema,’ **(J–L)** – *rhp1*.b mutant. Bars: **(A,D,G,J)** – 1 mm, **(B,E,H,K)** – 100 μm, **(C,F,I,L)** – 20 μm; **(A,D,G,J)** – Stereoscopic microscope images, **(B,C,E,F,H,I,K,L)** – Scanning Electron Microscope images.

The barley varieties Dema and Karat and their respective mutant lines *rhp1.b* and *rhl1.a* were selected as ideal candidates for this study due to the high variation in root hair development and structure between genotypes. The *rhp1.b* and *rhl1.a* genotypes were obtained from the chemical treatment of Dema and Karat, respectively (Janiak and Szarejko, 2007). The root hairs of *rhp1.b* develop to the primordium stage only and their tip growth is arrested after the bulge formation. Conversely, *rhl1.a* is a completely hairless mutant that exhibits a disturbed pattern of root epidermis cells, with undistinguishable trichoblasts (Marzec et al., 2013). The *rhp1.b* and *rhl1.a* are recessive mutations, have a monogenic inheritance and the genes responsible for the mutant phenotypes were mapped on barley chromosomes 1H and 7H, respectively (Chmielewska et al., 2014). In this study, we have used mutant lines that were backcrossed twice with their corresponding wild-type genotype and further self-pollinated seven times. **Figure 1** provides an overview of the root hair morphology of the genotypes used in this study.

Barley seeds were surface sterilized following established protocols (Bulgarelli et al., 2015) and germinated on 0.5% water agar plates. Seedlings displaying comparable rootlet development were sown individually in 12 cm diameter pots pre-filled with agricultural soils (see below). Plants were grown in a randomized design in a glasshouse at 18/14°C (day/night) temperature regime with 16 h day length daylight that was supplemented with artificial lighting to maintain a minimum light intensity of 200 μmol quanta m^-2^ s^-1^. Watering was conducted every 2 days with the application of 50 ml of deionized water to each pot. In total, five replicates of each barley line (i.e., five individual pots) were maintained alongside five unplanted pots containing the same soil substrates used as ‘Bulk’ soil controls. In addition, we maintained five plants derived from unsterilized seeds, not showing obvious symptoms of pathogen infection, to monitor the impact of seed sterilization on rhizosphere microbiota recruitment (Supplementary Database S1). Individual replicated pots were maintained in the glasshouse for 4 weeks post-transplantation, when the tested genotypes reached early stem elongation, corresponding to Zadoks stages 30–35 (Tottman and Makepeace, 1979).

The soil ‘Quarryfield’ was collected near the village of Kingoodie, Scotland, United Kingdom (56° 27′ 5′′ N, 3° 4′ 29′ W) while the soil ‘Tayport’ was sampled near the village of Tayport, Scotland, United Kingdom (56° 25′ 40′′ N, 2° 52′ 58′′ W). The physical and chemical properties of both substrates were determined using the soil analysis service of Yara United Kingdom Ltd. (Grimsby, United Kingdom) and are summarized in Supplementary Table S1.

### Bulk Soil and Rhizosphere DNA Preparation

At early stem elongation plants were excavated from the soil and the roots separated from the stems (Supplementary Figure S1). Stems were dried at 70°C for 48 h and the dry weight recorded. The roots were vigorously hand shaken to remove loosely adhering soil particles. For each plant, the uppermost 6 cm of the root system and its tightly adhering soil layer, which we operationally defined as the rhizosphere, were collected and placed in sterile 50 ml falcon tubes containing 15 ml Phosphate-buffered saline (PBS) solution. The samples were then vortexed for 30 s in order to dislodge and suspend the rhizosphere from the root. Using sterile forceps, the roots were then transferred to a second 50 ml falcon containing 15 ml PBS solution and vortexed for a second time in order to maximize the removal of rhizosphere substrate. Following this, the roots were removed and the PBS buffers were pooled together into an individual falcon tube and then centrifuged at 1,500 ×*g* for 20 min to collect the rhizosphere soil into a visible pellet. The supernatant was then discarded, and the sample frozen using liquid nitrogen and stored at -80°C for future DNA extraction.

Bulk soil samples were taken by removing a portion of the soil from the unplanted pots corresponding to the area explored by the roots in the planted samples and subjected to the same process as outlined above.

DNA was extracted from samples using FastDNA SPIN Kit for Soil (MP Biomedicals, Solon, United States) following the manufacturer’s instructions. Approximately 0.5 g of frozen bulk and rhizosphere samples were resuspended in the Sodium Phosphate and MT buffers, transferred into the Lysis Matrix E tubes and homogenized using the Tissue Lyser II instrument (Qiagen, Hilden, Germany) at 20 rotations s^-1^ for 30 s. DNA samples were eluted in 100 μl DES water and their concentrations were determined using the NanoDrop 1000 Spectrophotometer (Thermo Scientific, Wilmington, United States). DNA samples were stored at -20°C for future use.

### 16S rRNA Gene Amplicon Sequencing

The amplicon libraries were generated via a selective PCR amplification of the hypervariable V4 region of the 16S rRNA gene using the PCR primers 515F (5′-GTGCCAGCMGCCGCGGTAA-3′) and 806R (5′-GGACTACHVGGGTWTCTAAT-3′). The PCR primer sequences were fused with Illumina flow cell adapter sequences at their 5′ termini and the 806R primers contained 12-mer unique ‘barcode’ sequences to enable the multiplexed sequencing of several samples (Caporaso et al., 2012). For each individual bulk and rhizosphere preparations, 50 ng of DNA was subjected to PCR amplification using the Kapa HiFi HotStart PCR kit (Kapa Biosystems, Wilmington, United States). The individual PCR reactions were performed in 20 μl final volume and contained 4 μl of 5X Kapa HiFi Buffer, 10 ng Bovine Serum Albumin (Roche, Mannheim, Germany), 0.6 μl of a 10 mM Kapa dNTPs solution, 0.6 μl of 10 μM solutions of the 515F and 806R PCR primers and 0.25 μl of Kapa HiFi polymerase. The reactions were performed in a G-Storm GS1 Thermal Cycler (Gene Technologies, Somerton, United Kingdom) using the following conditions: 94°C (3 min), followed by 35 cycles of 98°C (30 s) denaturing, 50°C (30 s) annealing, 72°C (1 min) elongation and a final elongation step of 72°C (10 min). For each 515F-806R primers combination a no template control (NTC) was subjected to the same process. To minimize potential biases originating during the PCR amplifications individual reactions were performed in triplicate. Furthermore, at least two independent sets of triplicate reactions per barcode were performed. Aliquots of individual replicates and the corresponding NTCs were inspected on 1% agarose gels prior to purification. Only samples (*a*) that displayed the expected amplicon size and (*b*) whose corresponding NTCs were not detectable on agarose gel were retained for further analysis.

Individual PCR replicates were pooled in a barcode-wise manner and purified using Agencourt AMPure XP kit (Beckman Coulter, Brea, United States) with a ratio of 0.7 μl AMPure XP beads per 1 μl of sample. Following purification, 3 μl of each sample was quantified using Picogreen (Thermo Fisher, United Kingdom) following the manufacturer’s recommendations. Once quantified, individual barcode samples were pooled in an equimolar ratio to generate amplicon libraries. All library QC and processing was carried out in the Genome Technology group, James Hutton Institute. Illumina-compatible library pools were quality checked using a Bioanalyzer (High Sensitivity DNA Chip; Agilent Technologies) and quantified using both Qubit and qPCR (Kapa Biosystems, Wilmington, United States). Denaturation and dilution was performed as recommended (Illumina guide 15039740 v01) using average (mean of Qubit and qPCR) concentration measurements. Amplicon libraries were supplemented with 15% of 4 pM phiX solution. High-quality libraries were run at 10 pM final concentration on an Illumina MiSeq system with paired-end 2 × 150 bp reads following established protocols for FASTQ file generation (Caporaso et al., 2012).

### OTU Table and Taxonomy Matrices Generation

We used QIIME, version 1.9.0 (Caporaso et al., 2010), to process the FASTQ files produced by the MiSeq machine. Unless otherwise specified, we adopted the default parameters. Forward and reverse read files from individual libraries were decompressed and merged using the command join_paired_ends.py, imposing a minimum overlap of 5 bp between reads. Demultiplexing of overlapping paired-end (PE) reads and quality filtering was performed using the command split_libraries_fastq.py, imposing a minimum PHRED score of 20. Only these high-quality PE reads were used to define Operational Taxonomic Units (OTUs) at 97% sequence identity. OTUs were identified using the ‘closed reference’ approach against the chimera-checked Greengenes database (DeSantis et al., 2006), version 13_5. OTU-picking was performed using the SortMeRNA algorithm (Kopylova et al., 2012). Singleton OTUs, i.e., OTUs accruing only a single sequencing read in the whole dataset, were filtered *in silico* and not retained for further analysis. The OTU tables obtained from the two independent sequencing runs were merged using the command merge_otu_tables.py. The merged OTU table was further processed *in silico* to deplete OTUs assigned to chloroplasts and mitochondria, likely reflecting cross-amplification of host-derived DNA. A taxonomy matrix, depicting the number of reads assigned to individual phyla, was generated using the command summarize_taxa.py. The merged OTU table and the taxonomy matrix were used in R for statistical analysis and figure preparation (see below).

### Data Analysis

Statistical analysis was performed in R. Unless otherwise specified, the depicted functions were retrieved from the default installation of R or the R package Phyloseq (McMurdie and Holmes, 2013).

For alpha-diversity calculations, the OTU table was rarefied at an even sequencing depth of 9,202 sequencing reads per sample resulting in 6,083 unique OTUs. Observed OTUs and the Shannon Index were computed with the function estimate richness. Data were visualized using the function ggplot from the package ggplot2. For each dataset, the normality of data distribution was assessed using the Shapiro–Wilk test. For dataset whose Shapiro–Wilk test yielded a *p*-value < 0.05 (the alpha level we imposed to infer whether the data tested were normally distributed), we first assessed the impact of the soil effect using the function wilcox.test. This analysis was then followed by a non-parametric analysis of variance using the functions kruskal.test and the posthoc.kruskal.dunn.test from the package PMCMR. Conversely, for datasets that displayed a normal distribution an analysis of variance was adopted.

To assess phyla differentially enriched between unplanted soil and rhizosphere samples we conducted an analysis of composition of microbiomes on phyla rarefied counts using the package ANCOM (Weiss et al., 2017).

For beta-diversity calculations and the identification of individual bacteria differentially accumulated between microhabitats and genotypes, count data were not rarefied. Low abundance OTUs were eliminated from the table if they did not have at least five counts in 20% of the samples used. This represents a modification of an abundance threshold previously adopted for rice and a comparable sequencing protocol (Edwards et al., 2015). We reasoned that adjusting this threshold to 20% of the samples would have allowed us to discard poorly reproducible OTUs and at the same time retain distinctive and unique features of the investigated genotypes (i.e., the number of replicates per individual genotype is 5 out of 25 samples sequenced in each soil type). For beta-diversity calculations the OTU counts were transformed to relative abundances using the function transform_sample_counts. Weighted Unifrac, which is sensitive to OTU relative abundance and phylogenetic assignment (Lozupone and Knight, 2005), and Bray–Curtis, sensitive to OTU relative abundance only, distances were calculated using the function ordinate. The partitioning of distance matrices among sources of variation was calculated using the function adonis from the package Vegan.

To identify individual bacteria differentially recruited between microhabitats (i.e., bulk and rhizosphere) and barley genotypes we implemented a differential analysis of the count data using negative binomial generalized linear models using the package DESeq2 (Love et al., 2014). Raw OTU count data and sample information were converted into a DESeq object using the function DESeqDataSetFromMatrix. Differential analysis was performed with the function DESeq on separated OTU tables according to the soil type used. In this analysis first we determined the number of OTUs enriched in and discriminating rhizosphere profiles from unplanted soil in each of the tested genotype (designated, ‘rhizosphere effect’). Next, we performed pair-wise comparisons between members of the same wild-type-mutant pair (i.e., Karat versus *rhl1*.a and Dema versus *rhp1*.b, respectively; designated ‘sample effect’). We defined as a robust signature of the barley genotype on the microbiota only an OTU that was identified as both (a) significantly enriched in one genotype (or in the other) and in the pair-wise comparisons and (b) significantly enriched in the rhizosphere of the same plant genotype compared to unplanted soil (i.e., the intersection ‘sample effect’ and ‘rhizosphere effect’).

To determine the number of OTUs assigned to a given order in the pair-wise comparisons between mutants and wild-type plants we used the function count from the package plyr. To determine the probability of randomly identifying 11 or more OTUs assigned to a specific order among bacteria differentially recruited between mutants and wild-type plants, we calculated a cumulative hypergeometric probability. Calculations were performed using the function phyper and taking into account the total number of OTUs assigned to a given order within the rhizosphere enriched OTUs (*m*) the number of rhizosphere enriched OTUs not assigned to that given order (*n*), and the total number of OTUs differentially recruited between genotypes (*k*).

Venn diagrams were generated using the package VennDiagram.

The script used to analyze the data and generate the figures of this study is available on GitHub at https://github.com/BulgarelliD-Lab/Barley-RHM.

## Results

### Soil Type Impacts on DNA Extractions, Sequencing Library Properties, and Plant Growth

**FIGURE 2:**
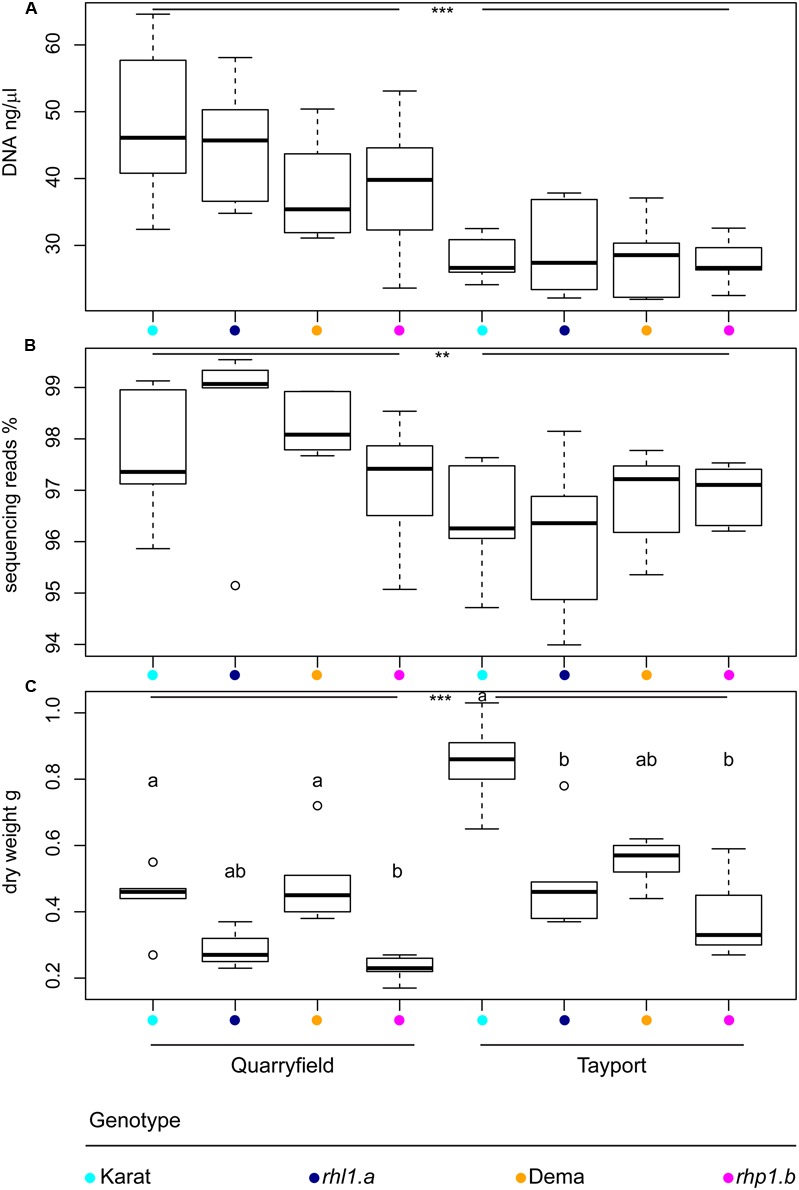
The soil type defines microbiota DNA properties and plant growth. Average **(A)** DNA concentration of the rhizosphere preparations, **(B)** proportion of sequencing reads assigned to microbial OTUs, and **(C)** above ground biomass of the indicated plant genotypes grown in Quarryfield and Tayport soils. Upper and lower edges of the box plots represent the upper and lower quartiles, respectively. The bold line within the box denotes the median. Maximum and minimum observed values are represented by the whiskers. Dots denote outlier observations whose value are 3/2 times greater or smaller than the upper or lower quartiles, respectively. Asterisks denote statistically significant differences between soil types (^∗∗∗^*P* < 0.001, ^∗∗^*P* < 0.01). In **(C)** different letters denote statistically significant differences between genotypes by Kruskal–Wallis non-parametric analysis of variance followed by Dunn’s *post hoc* test (*P* < 0.05, BH corrected).

We initially investigated the impact of soil type and the host genotype on the barley rhizosphere microbiota by inspecting the total amount of DNA recovered from our preparations. We observed that Quarryfield preparations yielded a significantly larger amount of DNA compared with samples obtained from Tayport soil (Mann–Whitney–Wilcoxon test, *P* < 0.001, **Figure 2A**). Conversely, when corrected for soil type, the host genotype did not impact on the total amount of DNA isolated from the rhizosphere preparations.

The amplicon sequencing of the 16S rRNA gene yielded a total of 5,847,887 high-quality sequencing reads. After *in silico* depletion of OTUs classified as Mitochondria or Chloroplasts, we reduced the total number of high-quality sequencing reads to 5,718,298. Therefore, we were able to retain for the downstream analysis nearly 98% of the original high-quality reads (mean per sample = 114,366 reads; max = 830,673 reads; min = 9,202 reads). Interestingly, we also observed a marked soil effect on the proportion of prokaryotic reads retrieved from rhizosphere samples. Congruently with the total DNA data, samples obtained from Quarryfield soil displayed a significantly higher proportion of reads classified as Bacteria and Archaea compared to Tayport soil and, even in this case, a host genotype effect was not detected (Two-way ANOVA; soil effect *P* < 0.01, genotype effect *P* = 0.77, Interaction term *P* = 0.30; **Figure 2B**).

We wondered whether these observations mirrored the growth performance of the plants in the two soils tested. Therefore, we measured stem dry weight at the time of sampling as a proxy for plant growth. Interestingly, the average biomass of plants grown in Tayport soil exceeded the data gathered for samples grown in Quarryfield soil (Mann–Whitney–Wilcoxon test, *P* < 0.05; **Figure 2C**), hence displaying an opposite trend compared to the microbial DNA data. However, once we performed pair-wise comparisons between the tested genotypes we failed to identify coherent patterns across soils. For instance, in Quarryfield soil, only the mutant *rhp1.b* displayed an above ground biomass significantly lower compared to the varieties Dema and Karat. Conversely, in Tayport soil, only the biomass of variety Karat exceeded that of both mutant genotypes (**Figure 2C**, Kruskal–Wallis and Dunn’s *post hoc* tests, *P* < 0.05, BH corrected).

### Bacteroidetes and Proteobacteria Dominate the Wild-type and Root Hair Mutants Rhizosphere Microbiota

**FIGURE 3:**
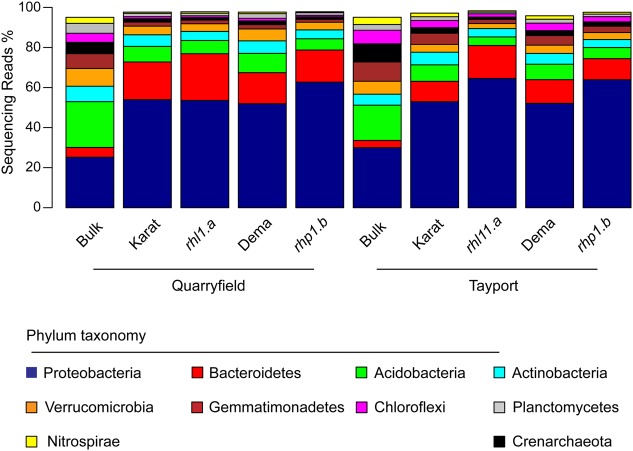
Bacteroidetes and Proteobacteria dominate the barley rhizosphere microbiota. Average relative abundance (% of sequencing reads) of the phyla identified in the indicated samples gathered from Quarryfield and Tayport soils. Only phyla displaying an average relative abundance > 1% in the whole sequencing dataset are included in the figure.

Overall, 45 bacterial and archaeal phyla were identified in our sequencing survey and after samples were rarefied at an even sequencing depth of 9,202 reads, 41 phyla were retained for downstream analysis (Supplementary Database S1). However, only 10 of these had a relative abundance greater than 1% and their aggregate mean relative abundance accounted for more than 97% of the generated sequencing reads (**Figure 3** and Supplementary Database S1). In both soils, and irrespective of the barley genotypes, rhizosphere specimens were significantly enriched in Bacteroidetes and Proteobacteria members: the average relative abundance of these two phyla in the rhizosphere reached 15.3 and 56.9%, a three- and two-fold increase, respectively, compared to bulk soil samples (ANCOM analysis, *P* < 0.05, FDR corrected, Supplementary Database S1). Conversely, members of the phylum Acidobacteria thrived less efficiently in association with barley roots: their average relative abundance declined from 20.2% in bulk soil to 6.9% in rhizosphere samples (Supplementary Database S1), whereas the proportion of other dominant phyla remained stable across sample type (e.g., Actinobacteria average relative abundance 6.6–5.1% across bulk and rhizosphere samples, respectively, Supplementary Database S1), although the fluctuations of these abundant phyla between unplanted soil and rhizosphere were not significant.

### Root Hair Mutants Host a Simplified and Distinct Rhizosphere Microbiota

**FIGURE 4:**
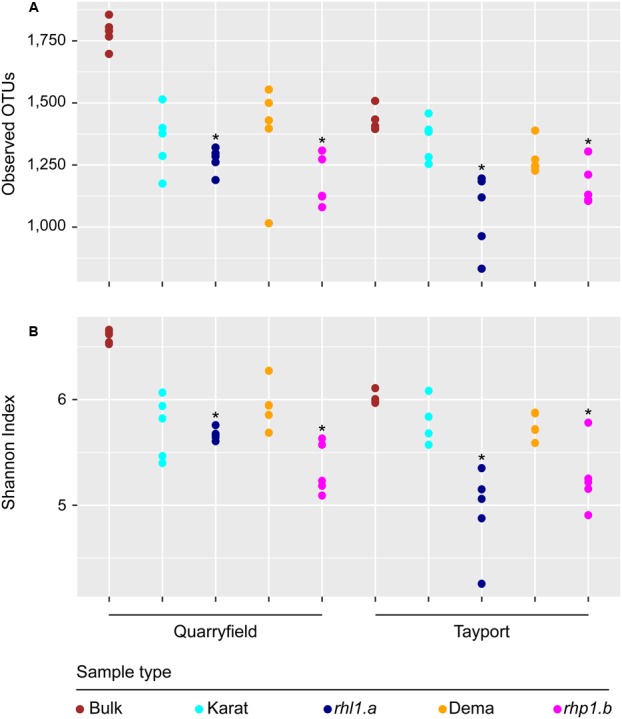
Root hair mutants host a ‘reduced-complexity’ community. **(A)** Total number of observed OTUs and **(B)** Shannon’s diversity index calculated for bulk and rhizosphere samples. Circles depict individual biological replicates. Asterisks denote statistically significant differences between the indicated samples and bulk soil controls based on Kruskal–Wallis non-parametric analysis of variance followed by Dunn’s *post hoc* test (*P* < 0.05, BH corrected).

We then analyzed within sample microbial diversity that is alpha-diversity. At the OTU level, we did not identify a significant effect of the soil type and the microhabitat on either the number of observed OTUs or the Shannon Index, proxies for bacterial richness and evenness, respectively (**Figure 4**, Mann–Whitney–Wilcoxon, *P* > 0.05). Conversely, we noticed a marked effect of root hair mutations: irrespective of the soil tested, the communities associated with *rhl1.a* and *rhp1.b* mutants were characterized by OTUs richness and evenness being significantly lower than the corresponding bulk soil samples (**Figure 4**, Kruskal–Wallis and Dunn’s *post hoc* tests, *P* < 0.05, BH corrected).

**FIGURE 5:**
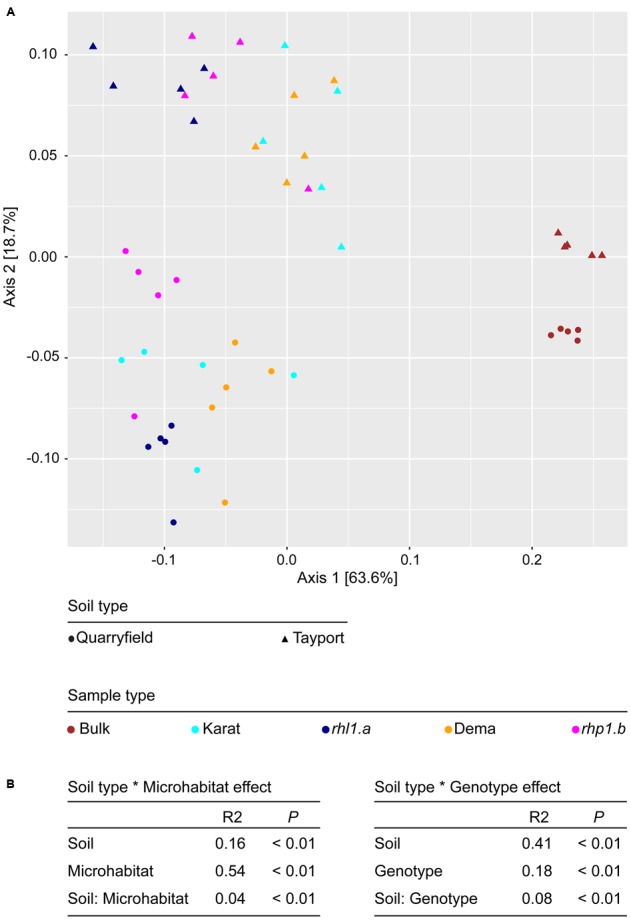
Root hair mutations do not impair the capacity of barley to shape the rhizosphere microbiota. **(A)** PCoA computed using the weighted Unifrac distance (sensitive to both OTU relative abundances and taxonomic affiliation). Replicates of bulk and rhizosphere samples are depicted by shapes whose spatial proximity reflects the degree of similarity of their microbiotas. **(B)** Permutational analysis of variances calculated using the Unifrac distance matrix for the indicated effects. The *R*^2^ values depict the proportion of variation in distances explained by the specified grouping of samples. Note that for the calculation of the Soil type ^∗^ Genotype effect, bulk soil samples were omitted from the analysis. *P*-values calculated for 5,000 permutations.

Subsequently, we characterized between samples diversity, which is beta-diversity. First, we adopted an abundance threshold to remove low-count, poorly reproducible OTUs from our analysis. Of the initial 8,811 OTUs identified only 1,993 passed our stringent abundance threshold. However, these retained OTUs accounted for 5,407,724 reads, i.e., more than 94% of the generated high-quality reads (Supplementary Database S1). Next, we used these data to generate weighted Unifrac and Bray–Curtis distance matrices. Using the weighted Unifrac distance, which is sensitive to OTU relative abundance and phylogenetic assignment, bulk soil and rhizosphere communities clearly segregated along the axis accounting for the largest variation in a Principal Coordinates Analysis, whereas the soil effect was efficiently recapitulated by the second axis (**Figure 5A**). This diversification was further supported by partitions of the distance matrix among sources of variation: the microhabitat had the strongest effect in determining the variation in the weighted Unifrac distances, followed by the soil effect and by their interaction term (**Figure 5B**). Interestingly, when we removed the bulk soil samples from the analysis and we reiterate the calculations, we identified a clear genotype dependent effect on the rhizosphere communities: the spatial separation between wild-type and mutants-associated communities observed in the PCoA (**Figure 5A**) was supported by a significant effect in the partitions of the variance (**Figure 4B**, Adonis test, genotype effect *R*^2^ = 0.18, *P* < 0.01), although this effect was significantly lower than the impact of the different soil type on the rhizosphere communities. When we performed the calculations using the Bray–Curtis distance matrix, which is sensitive to OTU relative abundance only, we observed a more pronounced impact of the soil on the barley microbiota. However, this marked effect did not mask the microhabitat and, most importantly, the genotype effects on the barley rhizosphere microbiota (Supplementary Figure S2).

### Individual Bacteria Discriminate between the Microbiotas of Wild-type and Root Hair Mutants

To identify bacteria whose presence and/or abundance supported the observed compositional diversification between microhabitats (i.e., the ‘rhizosphere effect’ on the microbiota), we determined the number of OTUs significantly enriched in and differentiating rhizosphere samples from unplanted soil. In Quarryfield soil, more than 500 OTUs fulfilled these criteria in each of the genotypes tested (Karat, number of rhizosphere enriched OTUs *n* = 537; Dema *n* = 529; *rhl1*.a *n* = 520; *rhp1*.b *n* = 557; Supplementary Database S1, Wald test, *P* < 0.01, FDR corrected). Likewise, and congruent with the separation observed in the PCoA plots, the analysis conducted in Tayport soil revealed that mutant plants enriched more OTUs in their rhizosphere compared to wild-type plants. (Karat, *n* = 452; Dema *n* = 473; *rhl1*.a *n* = 568; *rhp1*.b *n* = 558; Supplementary Database S1, Wald test, *P* < 0.01, FDR corrected). Thus, not only root hair mutants retained the capacity to shape the soil biota in a manner compared to wild-type plants, in at least one soil type (Tayport) their distinct profiles are represented by a greater number of significantly enriched OTUs.

**FIGURE 6:**
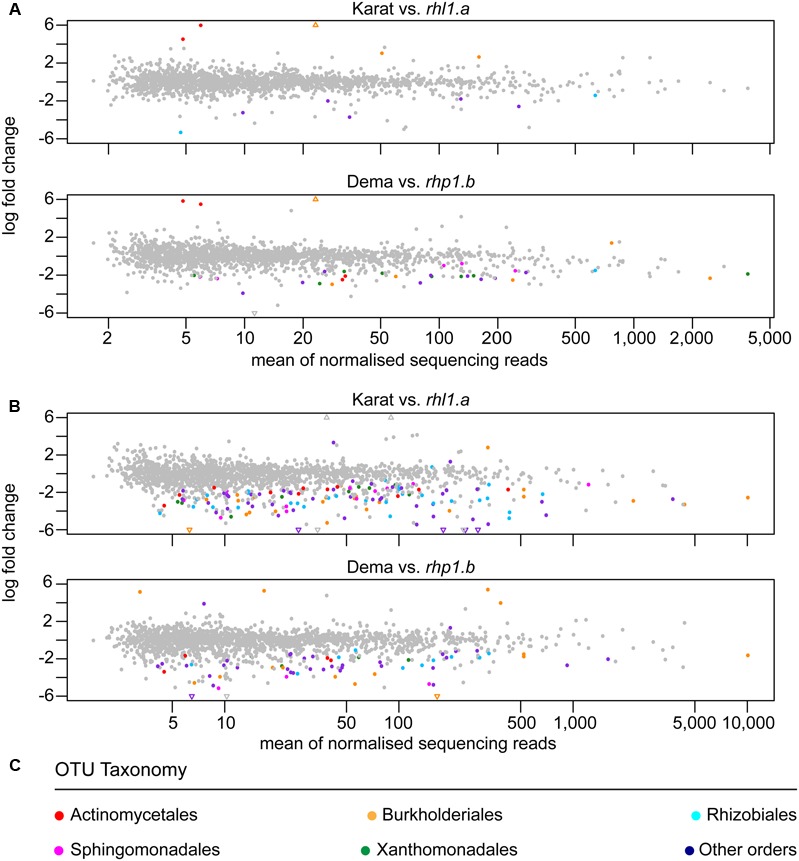
Individual bacteria discriminate between the microbiotas of wild-type and root hair mutants. OTUs differentially enriched in the indicated pair-wise comparisons between genotypes grown in **(A)** Quarryfield soil or **(B)** Tayport soil. Individual OTUs are depicted by circles whose *x*–*y* coordinates are defined by the mean abundance and the logarithmic fold change between wild-type and mutant genotypes, respectively. Triangles represent OTUs whose fold change exceeds the scale on the *y*-axis. In all the comparisons the positive fold change is associated with the enrichment of an OTU in wild-type specimens. OTUs significantly enriched (Wald test, *P* < 0.01, FDR corrected) are denoted by colors recapitulating **(C)** their taxonomic classification at the order level. The gray color indicates OTUs not significantly enriched.

Next, we investigated whether any of these rhizosphere-enriched OTUs discriminated between barley genotypes. In Quarryfield soil, the comparison Karat-*rhl1.a* yielded a total of 12 differentially enriched OTUs between genotypes, while 33 OTUs were identified as differentially regulated in the Dema-*rhp1.b*. These OTUs were not equally distributed between the terms of the comparison, rather, the majority of these OTUs, 7 and 29, were enriched in *rhl1.a* and *rhp1.b*, respectively (**Figure 6A**, Supplementary Database S1, Wald test, *P* < 0.01, FDR corrected). Interestingly, Tayport soil, which is characterized by a limited organic matter content compared to Quarryfield (Supplementary Table S1), triggered a more pronounced genotype effect on the barley microbiota. For instance, the comparison Karat-*rhl1.a* yielded a total of 132 differentially recruited OTUs between genotypes, whereas 70 OTUs differentiated between Dema and *rhp1.b* profiles (**Figure 6B** and Supplementary Database S1, Wald test, *P* < 0.01, FDR corrected). Strikingly, also in this soil type, the vast majority of these differentially recruited OTUs, 128 and 64, were significantly enriched in the mutant genotypes *rhl1.a* and *rhp1.b* compared to their cognate wild-type background (Karat and Dema, respectively). Of note, the impact of root hair mutations on the microbiota exceeded the effect of other or additional host genetic traits differentiating the tested genotypes. In particular, only 1 and 22 OTUs were identified as differentially recruited between wild-types Karat and Dema in Quarryfield and Tayport soils, respectively (Supplementary Database S1, Wald test, *P* < 0.01, FDR corrected).

**FIGURE 7:**
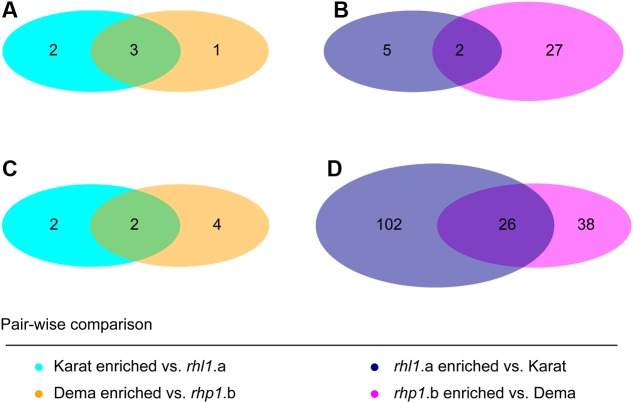
Soil type modulates the host genotype effect on the rhizosphere microbiota. Number of OTUs differentially recruited between genotypes grown in **(A,B)** Quarryfield soil or **(C,D)** Tayport soil. Diagram color depicts the indicated pair-wise comparisons. Intersections of the diagrams highlight OTUs differentially recruited in a soil type-independent manner. (Wald test, *P* < 0.01, FDR corrected).

When we inspected the taxonomic assignment of these OTUs, we noticed that members of the orders Actinomycetales, Burkholderiales, Rhizobiales, Sphingomonadales, and Xanthomonadales occurred in more than 10 instances in at least one soil type (**Figure 6C** and Supplementary Database S1). Cumulative hypergeometric calculation revealed that 11 or more occurrences of members of these orders among bacteria differentially recruited between wild-type and mutant plants have probabilities exceeding 0.75 (Supplementary Database S1). Considering that Actinomycetales, Burkholderiales, Rhizobiales, Sphingomonadales, and Xanthomonadales rank at the top in term of number of OTUs assigned among the 26 orders differentiating between wild-type and root hair mutants, our data suggest that the host genotype acts predominantly on abundant members of the microbiota. Interestingly, we did not detect a consistent overlap between the numbers of OTUs differentially recruited in wild-type and mutant plants in the two tested soils (**Figure 7**). A remarkable exception to this general trend was represented by an individual OTU, classified as *Janthinobacterium* sp., enriched in the mutant *rhp1.b* in a soil-independent manner and representing 6–12% of the entire rhizosphere community (Supplementary Database S1).

## Discussion

Despite the observed perturbations in rhizosphere formation provoked by root hair mutations (Brown et al., 2012; George et al., 2014), our data suggests that fully developed root hairs are not necessary for microbial proliferation at the barley root–soil interface (**Figure 2**). Rather, the major impact on microbial DNA was clearly exerted by the soil type, reinforcing the notion that edaphic factors drive the bacterial microbiota thriving at the root–soil interface (Bulgarelli et al., 2013). However, it is important to note that edaphic factors can interfere with the efficiency of DNA recovery *per se* rather than directly influencing microbial activity and biomass. For instance, specimens grown in the Tayport soil were associated with a significantly smaller amount of microbial DNA compared with samples obtained from Quarryfield soil, whose organic matter content is almost twice as much as the former substrate (organic matter Tayport 2.9%; Quarryfield 5.5%, Supplementary Table S1).

In turn, edaphic factors have repercussions on the growth performance of the plants: Tayport samples yielded a significantly higher aboveground biomass compared with Quarryfield samples. These observations prompted us to speculate that both the quantitative and qualitative (see below) nature of the rhizosphere microbiota draw upon photosynthetic resources of the plants to ensure optimum growth of the host in a given soil type.

In particular, the disproportion of Bacteroidetes and Proteobacteria in rhizosphere samples compared to bulk soil (**Figure 3**) is reminiscent of the selective bacterial enrichment observed for other cereal species, such as wheat (Turner et al., 2013), maize (Peiffer et al., 2013), and rice (Edwards et al., 2015). Remarkably, these recruitment profiles are comparable to the ones retrieved from wild and cultivated barley genotypes grown in a German agricultural soil (Bulgarelli et al., 2015), possibly representing a feature of the barley microbiota conserved across soil types. Together, these data indicate that, at a higher taxonomic rank, the recruitment of a distinctive rhizosphere microbiota is virtually unaffected by the root hair mutations characterized in this study.

Yet, when we increased the taxonomic resolution of our investigation to the OTUs level, we identified clear signatures of root hair development on microbiota recruitment. Remarkably, root hair mutants hosted a reduced-complexity microbiota compared with their cognate wild-type parents in both soil types. This was manifested in a significant reduction of ecological indices recapitulating richness and evenness of the communities (**Figure 4**). Of note, the ‘reduced-complexity’ communities inhabiting the rhizosphere of root hair mutants were clearly distinct from both the corresponding wild-type and bulk soil profiles (**Figure 5**), suggesting that these communities are the likely result of a perturbation of the host recruitment signals rather than an opportunistic colonization by the soil biota. Indeed, the distinctive characteristic of the root hair mutant microbiota was represented by the significant enrichment of individual bacteria, which ultimately dominated their rhizosphere profiles (**Figure 6**).

Two striking features were associated with these selective enrichments. The first one was a marked soil-dependency of this trait. Plants grown in Tayport soil yielded the highest number of differentially regulated OTUs compared to Quarryfield samples. In addition, these OTUs were not largely conserved across soil type (**Figure 7**). We previously demonstrated that a cooperative action of both host–microbe and microbe–microbe interactions shape the barley rhizosphere microbiota (Bulgarelli et al., 2015), we therefore speculate that the reduced amount of organic matter, and consequently a reduced microbial functional diversity, in Tayport soil has shifted the balance of this cooperative action in favor of the host recruitment cues. In addition, our results suggest that edaphic factors differentiating between the two soil types (Supplementary Table S1) promote the establishment of a microbiota whose metabolic capacities are better adapted to, or required by the host plant in a given soil type. The second striking feature is the fact that, in at least one of the soils tested, the mutant *rhp1.b* (extremely short root hairs) hosted a more distinct profile compared to the hairless mutant *rhl1.a* and wild-type plants with fully developed root hairs (**Figure 5**). This observation suggests that root hair length *per se* is not sufficient to explain the diversification of the microbiota observed between the tested genotypes.

A previous molecular characterization of the genetic pairs Karat-*rhl1.a* and Dema-*rhp1.b*, which identified a subset of proteins differentially accumulated in these genotypes (Janiak et al., 2012), might offer a direct link to the recruitment cues of the barley microbiota perturbed by root hair mutations. In particular, among the proteins identified in the aforementioned study, several ATP-binding cassette (ABC) transporters were identified. ABC transporters play a critical role in the secretion of phytochemicals during rhizodeposition (Baetz and Martinoia, 2014). Interestingly, the disruption of a specific ABC transporter, designated *abcg30*, altered both the exudate and microbiota profiles of *Arabidopsis thaliana*, although this effect was observed only in plants grown for two consecutive generations in the same soil (Badri et al., 2009). It is of note that this latter study further revealed that the major effect on the root-associated communities was the enrichment, in the mutant plants, of individual members of the microbiota. This is strikingly similar to the effect of the root hair mutations we observed in our study (**Figure 6**). We therefore hypothesize that ABC transporter-mediated modifications of rhizodeposition represents, at least in part, the perturbation of the host recruitment signals giving rise to an altered rhizosphere microbiota in root hair mutants. Further studies aimed at deciphering the impact of root hairs (or the lack thereof) on barley rhizodeposition will contribute to test these hypotheses.

Although the impact of root hair mutations on the rhizosphere microbiota is clearly modulated by the soil type at the highest resolution (i.e., the OTUs level), we identified a bias for members of the orders Actinomycetales, Burkholderiales, Rhizobiales, Sphingomonadales, and Xanthomonadales among the bacteria differentially recruited between wild-type and root hair mutants, which represent also the dominant members of the community (**Figure 6**).

Members of the aforementioned orders have previously been reported as Plant Growth Promoting Rhizobacteria (PGPRs) (Lugtenberg and Kamilova, 2009; Schlaeppi and Bulgarelli, 2015). Considering the critical role of root hairs as a site for nutrient acquisition in barley, it is tempting to hypothesize that an impairment of their development may trigger the recruitment of a specific microbiota capable of compensating for these limitations. This scenario is in agreement with the one proposed for arbuscular mycorrhizal fungi, whose colonization of barley roots is promoted in the hairless genotype, but not in mutants with short root hairs or wild-type plants, by phosphorus deficiency (Brown et al., 2013). Interestingly, the proliferation of rhizosphere-inhabiting *Janthinobacterium* spp., the dominant bacteria in *rhp1.b* community, has previously been reported as a distinctive feature of the bacterial communities associated with the extra radical mycelium of arbuscular mycorrhizal fungi (Scheublin et al., 2010) and promoted by fungal pathogens (Chapelle et al., 2016). Thus, the effect of root hair mutations on the composition of the rhizosphere bacterial microbiota could be, at least in part, the consequence of a perturbation of the symbiotic interactions between plant roots and soil fungi.

However, it is worth mentioning that when barley plants are grown under soil stress conditions, the yield of root hair mutants, although of different genetic backgrounds compared to the ones characterized in our study, is significantly lower compared with the one of wild-type plants (Brown et al., 2012; George et al., 2014). Therefore, an alternative hypothesis is that the differential recruitment we observed in our experiments reflects a dysbiosis of the barley microbiota *per se*. For instance, barley plants grown under phosphate-limiting conditions, a stress condition that could be triggered by an impairment of root hair functionality, display a shift in the exudation profile of organic acids (Wang et al., 2015). Organic acids and other low molecular weight compounds usually found in root exudates can shift microbiota composition in different soil types (Eilers et al., 2010). Consequently, mutant plants, through perturbed rhizodeposition, might recruit a ‘stress-induced microbiota.’ In particular, the distinct composition of the root hair mutant microbiota (**Figures 4**–**7**) might mirror an impairment of host-microbiota interactions needed for optimum plant growth under limiting nutrient supplies.

These two hypotheses are not necessarily mutually exclusive: the stress-induced microbiota may be represented by an increased proportion of PGPRs whose metabolic capacities cannot compensate for the root hair mutations. To disentangle the molecular relationships between root hairs, bacteria and other members of the rhizosphere microbiota, we can now deploy our sequencing data to assist with the systematic *in vitro* isolation of members of the barley microbiota. This, coupled with recolonization assays, will allow us to ultimately elucidate the impact of specific structural and functional configurations of the rhizosphere microbiota on given plant phenotypes.

## Accession Numbers

The sequences generated in this study are deposited in the European Nucleotide Archive (ENA) under the accession number PRJEB15634.

## Author Contributions

SR-A and DB conceived of and designed the experiments. AJ and IS provided access to the root hair mutants. TG provided access to the Tayport soil. SR-A, RAT, and KB performed the experiments. BC and JK generated the microscopic images of wild-type and root hair mutant plants. JM and PH generated the sequencing information. SR-A, MB, and DB analyzed the data. SR-A, AJ, PH, TG, and DB wrote the manuscript. All authors discussed the results and commented on the manuscript.

## Conflict of Interest Statement

The authors declare that the research was conducted in the absence of any commercial or financial relationships that could be construed as a potential conflict of interest.
