# Supplementary material for: Root Hair Mutations Displace the Barley Rhizosphere Microbiota
Source: Front Plant Sci. 2017 Jun 26;8:1094. doi: 10.3389/fpls.2017.01094 (PMC5483447; doi:10.3389/fpls.2017.01094)
Supplement: Supplementary file 2 [file Data_Sheet_2.PDF]

Supplementary Material

Root hair mutations displace the barley rhizosphere microbiota

Senga Robertson-Albertyn, Rodrigo Alegria Terrazas, Katharin Balbirnie, Manuel Blank, Agnieszka Janiak, Iwona Szarejko, Beata Chmielewska, Jagna Karcz, Jenny Morris, Pete E. Hedley, Timothy S. George and Davide Bulgarelli*

*** Correspondence:** d.bulgarelli@dundee.ac.uk

1 Supplementary Data

Supplementary Database 1 contains the following information:

Worksheet ws1: *Map_JH02_JH03_RHM_phyloseq.txt*. This is the design file of the experiment;

Worksheet ws2: *JH02_JH03_RHM_otu_table_nc2.txt*. This is the OTU count table generated in QIIME;

Worksheet ws3: *JH02_JH03_RHM_noPlant_OTUs_id.txt.* The OTU ids after the in silico depletion of OTUs classified as either Chloroplast or mitochondria;

Worksheet ws4: *JH02_JH03_RHM_L2_visualisation.txt*. This is the rarefied Phylum-level count matrix generated in QIIME where counts are collapsed for sample type;

Worksheet ws5: *JH02_JH03_RHM_otu_table_nc2_noPlants_L2_rare_sample.txt.* This is the rarefied Phylum-level count matrix generated in QIIME used for statistical analysis in R.

Worksheet ws6: *OTU_table_transposed.txt.* This *is the rarefied Phylum-level count matrix generated in QIIME and converted in 'wide format'.*

Worksheet ws7: This is the result of the ANCOM analysis.

Worksheet ws8: *JH02_JH03_RHM_dat_tax_noPlants_ordered.txt.* This is file contains the taxonomy information where individual ranks are organised column-wise of the OTUs identified in the survey after the in silico depletion of OTUs classified as either Chloroplast or mitochondria;

Worksheet ws9: *JH02_JH03_RHM_data_phyloseq_rare_table_counts_2.txt.* This is the OTU count table rarefied at even sequencing depth for alphadiversity calculation.

Worksheet ws10-13: Statistical and taxonomic information of the OTUs significantly enriched in and discriminating between rhizosphere and unplanted soil samples in the genotypes Karat, *rhl1*.a, Dema and *rhp1*.b, respectively (Quarryfield soil data);

Worksheets ws14-17: Statistical and taxonomic information of the OTUs differentially recruited in the pair-wise comparisons: Karat enriched vs. *rhl1.a*; *rhl.1a* enriched vs. Karat; Dema enriched vs. *rhp1.b*; *rhp1.b* enriched vs. Dema, respectively (Quarryfield soil data);

Worksheet ws18-21: Statistical and taxonomic information of the OTUs significantly enriched in and discriminating between rhizosphere and unplanted soil samples in the genotypes Karat, *rhl1*.a, Dema and *rhp1*.b, respectively (Tayport soil data);

Worksheets ws22-25: Statistical and taxonomic information of the OTUs differentially recruited in the pair-wise comparisons: Karat enriched vs. *rhl1*.a; *rhl1*.a enriched vs. Karat; Dema enriched vs. *rhp1*.b; *rhp1*.b enriched vs. Dema, respectively (Tayport soil data);
Worksheets ws26-27: Statistical and taxonomic information of the OTUs differentially recruited in the pair-wise comparisons: Karat enriched vs. Dema; Dema enriched vs. Karat respectively (Tayport soil data);
Worksheet ws28: Cumulative hypergeometric probabilities computed for Actinomycetales, Burkholderiales, Rhizobiales, Sphingomonadales and Xanthomonadales.

2 Supplementary Figures and Tables

	Quarryfield	Tayport
**Organic matter (%)**	5.0	2.9
**Soil particles (%)**		
**Silt**	47.39	39.95
**Clay**	11.28	12.29
**Sand**	41.33	48.36
**Soil texture**	Sandy Silt Loam	Sandy Silt Loam
**pH**[1]	6.2	6.2
**Mineral content (ppm)**		
**Phosphorus**[2]	99	101
**Potassium**	162	429
**Magnesium**	156	292
**Calcium**	2,661	1,786

Supplementary Table 1. Chemical and physical characteristic of the agricultural soils used in this study. 1-Determined using water as extractant. 2-Determined using the Olsen method.

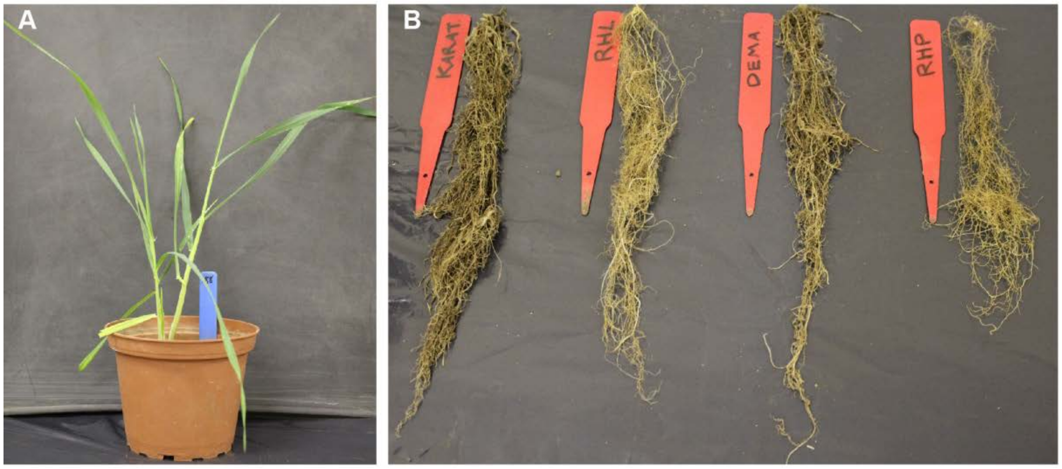

Figure S1 Representative above- and below-ground characteristics of the plants used in this study. **(A)** Barley plants at the time of sampling **(B)** Whole root specimens of the indicated genotypes with adhering rhizosphere soil. Label width = 1.5 cm; Label length = 13 cm.

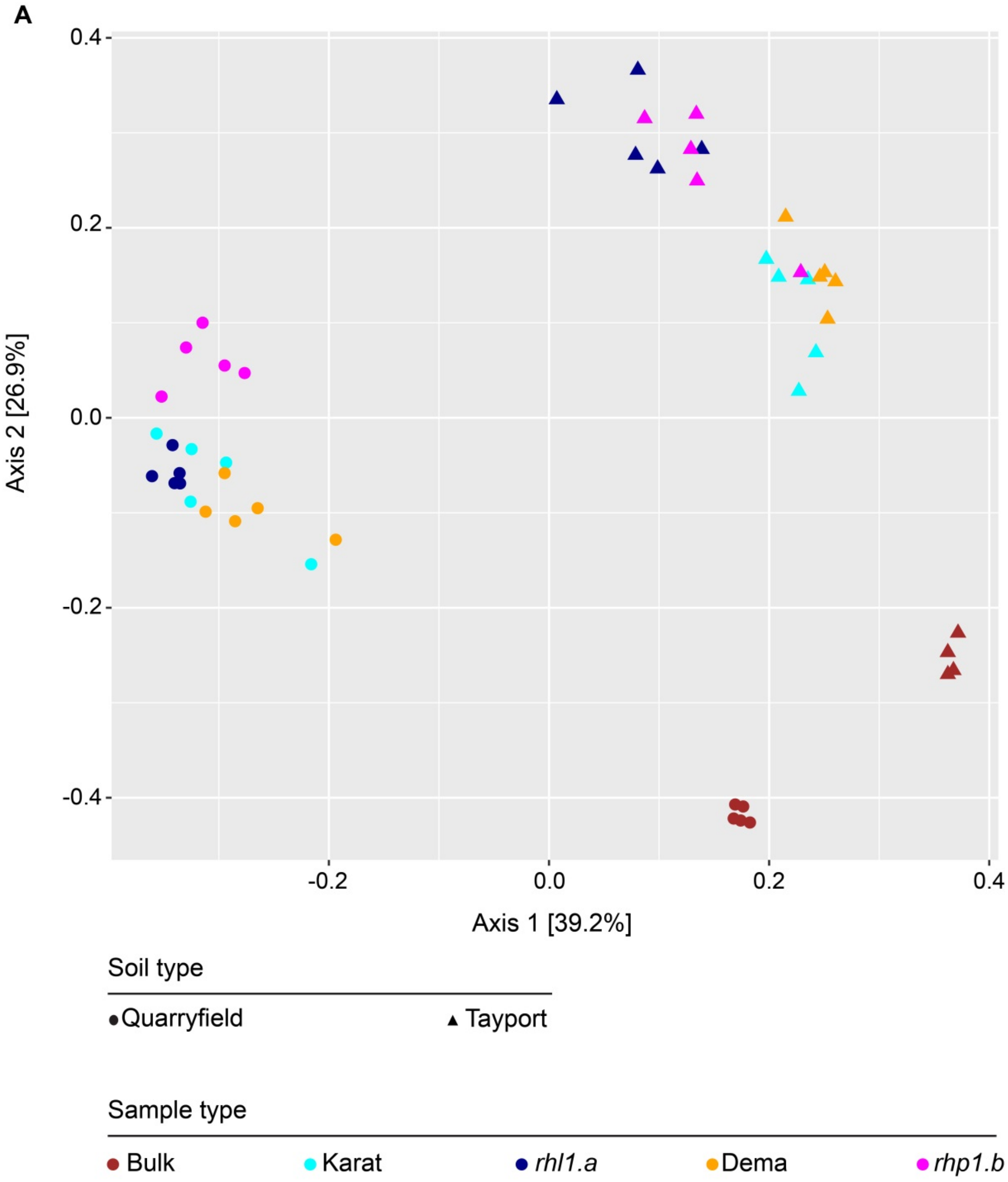

B

Soil type * Microhabitat effect

	R2	*P*
Soil	0.33	< 0.01
Microhabitat	0.27	< 0.01
Soil: Microhabitat	0.06	< 0.01

Soil type * Genotype effect

	R2	*P*
Soil	0.51	< 0.01
Genotype	0.12	< 0.01
Soil: Genotype	0.08	< 0.01

Figure S2 Root hairs fine-tune the composition of the rhizosphere microbiota in a soil-dependent manner. (**A**) PCoA computed using the Bray-Curtis distance (sensitive to OTU relative abundances). Replicates of bulk and rhizosphere samples are depicted by shapes whose spatial

proximity reflects the degree of similarity of their microbiotas. (**B**) Permutational analysis of variances calculated using the Bray-Curtis distance matrix for the indicated effects. The R-squared values depict the proportion of variation in distances explained by the specified grouping of samples. Note that for the calculation of the Soil type * Genotype effect, bulk soil samples were omitted from the analysis. *P* values calculated for 5,000 permutations.
